# Targeting toll-like receptors: unveiling potential therapeutic strategies for deep vein thrombosis

**DOI:** 10.3389/fimmu.2025.1579113

**Published:** 2025-05-29

**Authors:** Wei Shao, Zilong Wang, Jian Wu, Tianting Guo, Jianwen Mo

**Affiliations:** ^1^ The First Clinical College, Gannan Medical University, Ganzhou, China; ^2^ Department of Orthopedics, The First Affiliated Hospital of Gannan Medical University, Ganzhou, China; ^3^ Department of Orthopedics, Ganzhou Hospital of Guangdong Provincial People’s Hospital, Ganzhou Municipal Hospital, Ganzhou, China

**Keywords:** deep vein thrombosis, toll-like receptors, inflammation, signal transduction, targeted therapy

## Abstract

Deep vein thrombosis (DVT) is a complex multifactorial vascular disease characterized by abnormal blood stasis and coagulation within the deep veins, primarily occurring in the lower limbs. This pathological condition not only causes local circulatory disruption but also has the potential to trigger life-threatening pulmonary embolism through thrombus detachment, thus posing a major threat to human health. Toll - like receptors (TLRs), essential components of the innate immune system, have been increasingly acknowledged as crucial determinants in the pathogenesis of DVT. TLRs possess the ability to recognize a diverse range of pathogen - associated molecular patterns (PAMPs) and endogenous danger - associated molecular patterns (DAMPs). Upon activation, they trigger a cascade of inflammatory responses that are intricately intertwined with the thrombotic process. This review comprehensively scrutinizes the extant knowledge pertaining to the role of TLRs in DVT. It systematically synthesizes the molecular mechanisms underpinning the participation of TLRs in DVT, spanning platelet activation, endothelial cell dysfunction, and leukocyte recruitment. Moreover, it delves profoundly into the potential of targeting TLRs as therapeutic strategies for DVT. This entails the exploration of the development and application of TLR inhibitors or antagonists. By elucidating these aspects, the objective is to proffer novel perspectives and insights for the prevention and treatment of DVT.

## 1 Introduction

DVT is a prevalent vascular condition that holds considerable clinical significance and exhibits a high occurrence rate, particularly in patients who have recently fracture, undergone surgery, individuals who remain immobile for extended periods, and those diagnosed with cancer (1). DVT not only causes lower limb swelling, pain, and functional impairment, but may also lead to serious complications such as pulmonary embolism, which can threaten patients’ lives (2). According to the latest epidemiological data, the incidence of DVT is on the rise globally, and its economic burden and social impact cannot be ignored (3). Data from a multinational cohort (2017–2023) involving over 1 million surgical patients revealed that DVT and pulmonary embolism (PE) accounted for 51% and 49% of venous thromboembolism (VTE) cases (4). Preoperative DVT prevalence in lower-extremity fractures reached 24.1%, with femoral fractures posing higher risks than tibiofibular injuries (5). In the pathophysiological process of DVT, Virchow’s triad—blood flow stasis, hypercoagulable state, and vascular endothelial damage—are considered the main pathogenic factors (6). However, research in recent years has shown that immune-inflammatory response plays a key role in the occurrence and development of DVT, especially the mediating effects of inflammatory cells and cytokines (7).

TLR, is widely present in various immune and non-immune cells. It plays a role in identifying PAMPs and DAMPs, which in turn triggers multiple signaling pathways and modulates immune responses (8). The activation of TLR signaling pathways is significant not only for anti-infection immunity but is also intricately connected to the development of various inflammatory diseases, such as atherosclerosis, rheumatoid arthritis, and DVT (9, 10). Recently, an increasing amount of research has concentrated on the particular mechanisms involved in TLR signaling pathways related to DVT, as well as their possible therapeutic targets. For example, the activation of TLR4 can lead to the activation of key transcription factors such as NF-κB and IRF3 through MyD88-dependent and MyD88-independent pathways, thereby inducing the expression of inflammatory factors and coagulation factors (11). In addition, TLR signaling pathways in endothelial cells have also been found to be closely related to vascular endothelial dysfunction, which in turn promotes thrombosis formation (12, 13). The results not only enhance our comprehension of how DVT develops but also offer a foundational theory for creating new treatment approaches.

In terms of treatment, the existing DVT treatment methods mainly include anticoagulant therapy and thrombolytic therapy. Although these methods have achieved certain effects in the prevention and treatment of thrombosis, they still have the risk of bleeding and therapeutic limitations (14–16). Therefore, the regulation of TLR signaling pathways has become a promising new therapeutic strategy. TLR antagonists and inhibitors have shown good antithrombotic effects in experimental models, indicating their potential for clinical application (17, 18). In addition, research on natural products has also shown that by regulating TLR signaling pathways, the inflammatory response and thrombosis formation of DVT can be effectively alleviated (19). This review aims to focus on the specific roles of TLR in the pathophysiological process of DVT, regulatory mechanisms, and therapeutic strategies based on TLR signaling pathways. By comprehensively analyzing the latest research progress, this article aims to provide new theoretical basis and research directions for the prevention and treatment of DVT.

## 2 The pathophysiological mechanisms of deep vein thrombosis

### 2.1 Virchow’s triad

DVT is a complex pathological process, the pathogenesis of which is mainly driven by Virchow’s triad, including blood flow stasis, hypercoagulable state, and vascular endothelial damage (6). Blood flow stasis is usually caused by venous valve dysfunction, long-term bed rest, or restricted activity after surgery, leading to a slowdown in venous blood flow and an increased risk of thrombosis (20, 21). Hypercoagulable state can be caused by hereditary coagulation factor abnormalities (such as prothrombin gene mutation, protein C/protein S deficiency) or acquired factors (such as cancer, pregnancy, oral contraceptives), which promote thrombosis by increasing the activity of coagulation factors or reducing the level of anticoagulant factors (22–24). Vascular endothelial damage promotes platelet adhesion and aggregation and initiates the coagulation cascade by exposing subendothelial matrix components (such as collagen) and procoagulant substances (such as tissue factor) (25–27).

### 2.2 The role of inflammatory response in DVT

**Figure 1:**
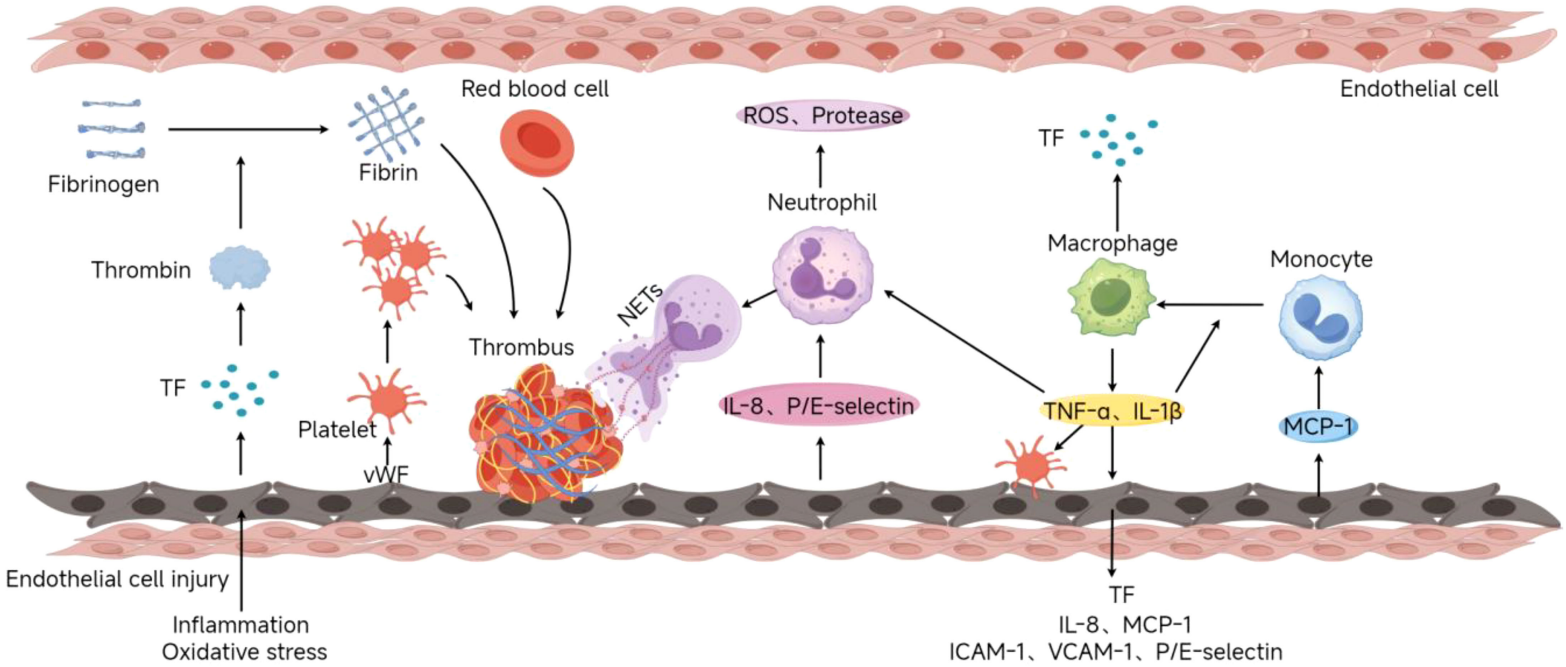
Deep Vein Thrombosis Formation Process(By Figdraw). Endothelial injury caused by inflammation or oxidative stress exposes the subendothelial matrix (e.g., collagen, vWF) and releases procoagulants like tissue factor (TF). Platelets bind to vWF via GPIb-IX-V, adhere to the site, and release mediators (ADP, TXA2) to recruit additional platelets, forming aggregates. TF activates the extrinsic coagulation pathway, generating thrombin to convert fibrinogen into fibrin mesh. Damaged endothelium releases chemokines (IL-8) and adhesion molecules (P/E-selectin), recruiting neutrophils that release NETs to stabilize thrombi. Monocytes recruited via MCP-1 differentiate into macrophages, secreting pro-inflammatory cytokines (TNF-α, IL-1β) and TF.

In addition to Virchow’s triad, the inflammatory response is also significant in the pathophysiology of DVT (**Figure 1**). Inflammatory cells, such as neutrophils, monocytes, and macrophages, regulate the processes of thrombosis and dissolution by releasing cytokines, chemokines, and enzymes (28–31). Neutrophils release Neutrophil Extracellular Traps (NETs), which not only capture pathogens but also promote thrombus stabilization and formation. NETs are composed of DNA, histones, and antimicrobial proteins, which can enhance fibrin formation and promote platelet activation and aggregation, thus accelerating thrombus development (32). Monocytes and macrophages further enhance the coagulation response by expressing tissue factor(TF) and other procoagulant factors in DVT. Studies have shown that monocytes accumulate in the venous intima and promote thrombus formation and maintenance by secreting TF and inflammatory mediators (such as IL-6, TNF-α) (33–35). In addition, macrophages participate in thrombus dissolution and fibrin degradation by clearing red blood cells and fibrin in the thrombus, maintaining the dynamic balance of the thrombus (36, 37).

## 3 Overview of TLR signaling pathways

### 3.1 Types and structures of TLR

Pattern recognition receptors (PRRs) of the innate immune system, known as TLRs, play a crucial role. TLRs were first discovered in fruit flies, and their name originates from the description of their importance by German scientists (“Toll” means “amazing” in German) (38). In 1997, the first Toll-like receptor, TLR4, was identified in mammals, marking the beginning of a new era in TLR research (39). In humans, 10 TLRs (TLR1 to TLR10) have been identified, while in mice, there are 12 (TLR1 to TLR9 and TLR11 to TLR13) (40). These receptors are distributed on immune cells (such as macrophages, dendritic cells, neutrophils, etc.) and some non-immune cells (such as endothelial cells, epithelial cells, etc.). They trigger complex signaling cascades by recognizing specific exogenous or endogenous molecules (41, 42).

TLRs constitute a group of transmembrane proteins characterized by a distinct molecular architecture featuring three components: the extracellular region, the transmembrane segment, and the intracellular signaling region. The extracellular domain of TLRs consists of multiple leucine-rich repeats (LRRs), which are arranged in a horseshoe shape to form specific ligand-binding sites (43). The extracellular domain configuration of various TLRs defines the specificity of their ligands (**Table 1**). For example, TLR4 exerts anti-Gram-negative bacterial effects by binding to lipopolysaccharide (LPS) (44). TLR9 recognizes CpG DNA (DNA sequences rich in unmethylated CpG dinucleotides), which is a characteristic of bacterial and viral DNA (45). These recognition capabilities make TLRs play a key role in anti-infection immunity and inflammatory responses, and they are also involved in the pathogenesis of various autoimmune and inflammatory diseases. The transmembrane domain of TLRs consists of a hydrophobic α-helical structure embedded in the cell membrane or endosomal membrane, serving to stabilize the receptor and mediate signal transduction (46). The transmembrane region not only aids in the localization of TLRs within the membrane but is also involved in the dimerization of receptors, a crucial step in the activation of signaling. The intracellular part of TLRs is called the Toll/IL-1 receptor domain (TIR), which is the core region for signal transduction (47). After ligand binding, the TIR domain interacts with specific adaptor proteins (such as MyD88 or TRIF) to activate downstream signaling pathways (48). The diversity of the TIR domain also supports the specificity of signal transduction by different TLRs.

**Table 1:** TLR ligands, sources, and functions.

TLR	Ligands	Sources	Functions
TLR1	Triacyl lipopeptides	Gram-positive bacteria, Mycobacteria	Enhances TLR2 recognition
TLR2	Lipoproteins, fungal components	Gram-positive bacteria, fungi, viruses	Broadly recognizes microbial components
TLR3	Double-stranded RNA (dsRNA)	Viruses	Antiviral immunity
TLR4	Lipopolysaccharides (LPS), viral proteins, endogenous molecules	Gram-negative bacteria, viruses, damaged cells	Initiates strong inflammatory responses
TLR5	Flagellin	Flagellated bacteria	Recognizes flagellated bacteria
TLR6	Diacyl lipopeptides	Gram-positive bacteria, Mycobacteria	Enhances TLR2 recognition
TLR7	Single-stranded RNA (ssRNA)	Viruses	Antiviral immunity
TLR8	Single-stranded RNA (ssRNA)	Viruses	Initiates inflammatory responses
TLR9	Unmethylated CpG DNA	Bacteria, viruses	Antimicrobial and antiviral immunity
TLR10	Unknown (possibly synergizes with TLR2)	Bacteria, fungi	Immune modulation (function not clear)
TLR11	Toxoplasma profilin, uropathogenic E. coli components	Toxoplasma, uropathogenic E. coli (mouse-specific)	Antiparasitic and antibacterial immunity
TLR12	Toxoplasma profilin	Toxoplasma (mouse-specific)	Antiparasitic immunity
TLR13	Bacterial 23S rRNA	Gram-positive bacteria (mouse-specific)	Antibacterial immunity

### 3.2 TLR signaling pathways

**Figure 2:**
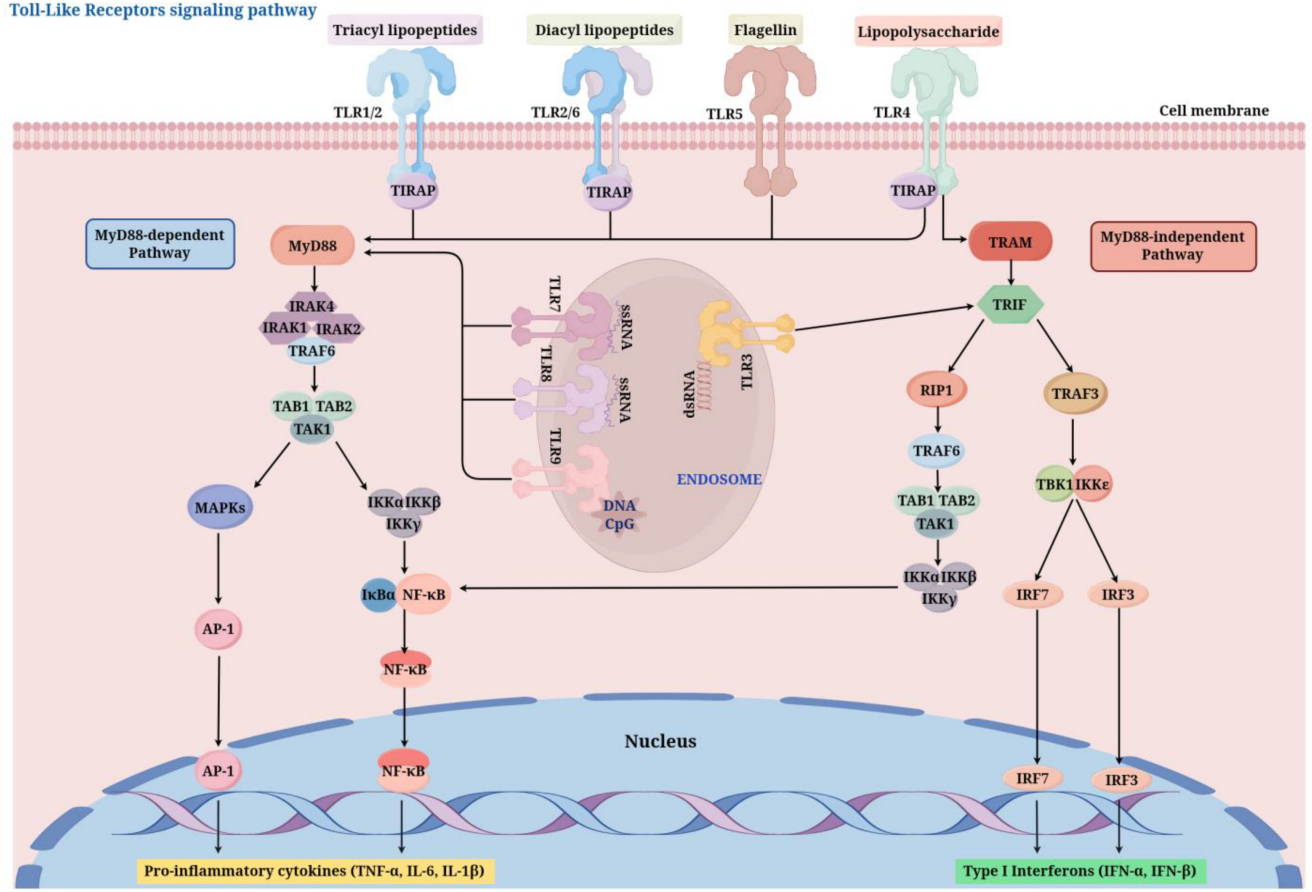
TLR signaling pathways (By Figdraw). After ligand binding (e.g., LPS for TLR4, dsRNA for TLR3), TLRs dimerize and recruit adaptor proteins such as MyD88 or TRIF through their TIR domains. The MyD88-dependent pathway activates IRAK and TRAF6, leading to NF-κB and MAPK activation, which induces pro-inflammatory cytokines. The MyD88-independent Pathway, utilized by TLR3 and TLR4, activates TBK1 and IRF3, promoting the production of type I interferons. TIRAP, TIR Domain-Containing Adaptor Protein; MyD88, myeloid differentiation factor 88; IRAK1/2/4, interleukin 1 Receptor Associated Kinase 1/2/4; TRAF, tumor necrosis factor receptor-associated factor ; TAB1/2, TGF-beta Activated Kinase 1 Binding Protein 1/2; TAK1, transforming growth factor activating kinase-1; NF-κB, nuclear factor kappa B; MAPK, mitogen-activated protein kinase; AP-1, Activator Protein 1; TRAM, TRIF-Related Adaptor Molecule; TRIF, toll receptor-associated interferon activator; RIP1, Receptor-Interacting Protein 1; IKKϵ, I-κB kinase ϵ; TBK1, TANK-binding kinase 1; IRF, interferon regulatory factor .

The activation of TLR signaling pathways mainly occurs through two different downstream pathways (**Figure 2**): the MyD88-dependent pathway and the MyD88-independent pathway (49). Most TLRs (except TLR3) activate downstream signaling molecules through the adaptor protein MyD88 (Myeloid differentiation primary response 88), this process activates the NF-κB (nuclear factor κB) and MAPK (mitogen-activated protein kinase) signaling pathways, leading to an increase in the production of pro-inflammatory cytokines, including TNF-α and IL-6 (50). TLR3 and TLR4, on the other hand, can use the adaptor protein TRIF (TIR-domain-containing adapter-inducing interferon-β) to activate IRF3 (interferon regulatory factor 3) and NF-κB, inducing the production of type I interferons (such as IFN-β) (51). Inside the cell, the activation process of TLR signaling pathways involves multiple key molecules and steps. First, after TLRs bind to their ligands, the receptors undergo dimerization or oligomerization, which then recruits adaptor proteins (such as MyD88, TRIF, TIRAP/MAL, TRAM) to the TIR domain of the receptor. These adaptor proteins further recruit downstream kinases (such as IRAK4, IRAK1) and E3 ubiquitin ligases (such as TRAF6), forming a signaling complex that promotes the activation of the IκB kinase (IKK) complex (52). The IKK complex adds phosphate groups to the IκB protein, resulting in its breakdown and the subsequent release of the NF-κB transcription factor into the nucleus, where it prompts the transcription of genes related to inflammation (53). Moreover, the stimulation of the MAPK signaling pathway increases the expression of inflammatory factors and other biological processes, such as cell growth and differentiation (54). In addition to the classical MyD88-dependent and -independent pathways, research in recent years has revealed the complexity and diversity of TLR signaling pathways. For instance, when LPS is recognized by TLR4, it not only activates NF-κB via the MyD88-dependent pathway but also triggers the expression of antiviral genes through the TRIF-dependent pathway, showcasing its diverse functions in combating infections (55). Moreover, the regulation of TLR signaling pathways is also influenced by various negative regulatory mechanisms, including the endocytosis of receptors, ubiquitination of adaptor proteins, and the involvement of negative regulatory proteins (such as A20, SARM). These mechanisms collectively maintain the balance of TLR signaling, preventing excessive inflammatory responses and the occurrence of autoimmune diseases (56, 57).

## 4 The role of TLR signaling pathways in DVT

### 4.1 TLR activation and thrombosis formation

TLR1 and TLR6 usually form dimers with TLR2, recognizing bacterial lipopeptides and other bacterial components, activating the MyD88-dependent signaling pathway, promoting the activation of NF-κB and MAPK, inducing the expression of inflammatory and coagulation factors, and thus promoting thrombosis formation (58). TLR3 mainly recognizes double-stranded RNA through the TRIF-dependent pathway, activating the IRF3 and NF-κB signaling pathways, inducing the production of type I interferons and pro-inflammatory cytokines, and enhancing the inflammatory environment for thrombosis formation (59).

One of the most thoroughly researched members of the TLR family is TLR4, which plays a crucial role in DVT and various other thrombotic conditions. This receptor is capable of identifying endogenous DAMPs like high-mobility group box 1 (HMGB1) as well as exogenous PAMPs such as bacterial LPS, activating downstream signaling through both MyD88-dependent and TRIF-dependent pathways, inducing the expression of inflammatory factors (e.g., TNF-α, IL-6) and coagulation factors (e.g., tissue factor), and promoting thrombus formation and stabilization (60, 61). Recent studies have shown that the activation of TLR4 also involves the interplay between the PI3K/Akt and MAPK signaling pathways, further enhancing inflammatory responses and thrombus stability (62–64).

TLR5 mainly recognizes bacterial flagellin, activating NF-κB and MAPK through the MyD88-dependent pathway, promoting the expression of inflammatory and coagulation factors, and thereby promoting thrombosis formation (65, 66). Despite receiving less attention, TLR7 and TLR8 have been demonstrated to contribute to the regulation of immune responses and coagulation processes in DVT. They mainly recognize single-stranded RNA, participate in inflammation associated with viral infections, and induce the production of type I interferons and pro-inflammatory cytokines through the IRF7 and NF-κB signaling pathways, promoting thrombosis formation (67, 68).

TLR9 recognizes CpG DNA, activating the IRF7 and NF-κB signaling pathways, inducing the production of type I interferons and pro-inflammatory cytokines, and further promoting thrombosis formation (69, 70). TLR10 is considered to have inhibitory functions in humans, negatively regulating the signaling pathways of TLR2 and TLR4 to limit excessive inflammatory responses and maintain vascular homeostasis (71–73). TLR11 to TLR13 in mice mainly participate in parasite infections and autoimmune regulation, and their specific roles in thrombosis formation require further research (74, 75).

### 4.2 Cellular sources and targets of TLRs in thrombosis

The role of TLRs in DVT depends not only on their expression levels but also on their distribution and functions in different cell types. The main TLR-expressing cells include endothelial cells, platelets, monocytes, macrophages and neutrophils.

#### 4.2.1 Endothelial cells

Vascular endothelial cells are crucial for sustaining vascular balance and managing the process of blood clotting. The activation of TLR signaling pathways can lead to endothelial cell dysfunction, promoting thrombosis formation. Specifically, the activation of TLR4 in endothelial cells induces the expression of inflammatory factors (e.g., IL-6, TNF-α) and adhesion molecules (e.g., VCAM-1, ICAM-1), enhancing the adhesion and migration of white blood cells, and promoting the accumulation and activation of inflammatory cells in the vascular wall (76, 77). Further studies have revealed that TLR4 regulates the microenvironment of endothelial cells, inducing oxidative stress and apoptosis, disrupting the integrity of the vascular endothelium, increasing the permeability of the vascular wall, and promoting thrombus formation and expansion (78). Additionally, TLR4 regulates the metabolic pathways of endothelial cells, promoting lipid peroxidation and the generation of inflammatory mediators, further exacerbating endothelial cell dysfunction (79, 80). The mechanisms of TLR2 and TLR9 in endothelial cells have also been gradually revealed. TLR2 regulates the proliferation and migration of endothelial cells through the PI3K/Akt and NF-κB signaling pathways, promoting vascular remodeling and thrombosis formation (81). TLR9 affects the ability of endothelial cells to cope with oxidative stress by regulating their antioxidant defense system, thereby influencing thrombosis formation (82). Research indicates that the relationship between TLRs and vascular endothelial cells extends beyond inflammatory responses, encompassing mechanisms associated with metabolism, cell viability, and apoptosis, all of which play a role in the emergence and progression of DVT.

#### 4.2.2 Platelets

Platelets are not only key participants in hemostasis and thrombosis formation but also play important roles in immune regulation and inflammatory responses. Recent research has shown that platelets regulate thrombo-inflammatory responses in various pathological conditions through TLR-mediated signaling pathways. TLR2 and TLR4 regulate platelet function through different mechanisms: the TLR2 agonist Pam3CSK4 directly induces platelet aggregation, while the TLR4 agonist LPS enhances platelet adhesion and glycoprotein Ib-dependent aggregation (83). In SARS-CoV-2 infection, TLR4 promotes platelet activation and thrombosis formation by binding to the viral spike protein, a mechanism of significant importance in COVID-19-related thrombotic complications (84). Furthermore, in individuals diagnosed with essential thrombocythemia (ET), there is a notable increase in the expression levels of TLR2 and TLR4, which contribute to the activation of platelets and the secretion of pro-inflammatory mediators through the activation of NF-κB and MAPK signaling pathways, thereby intensifying thrombo-inflammatory reactions (85). Calprotectin induces platelet apoptosis by binding to TLR4 on the platelet surface and activating the NLRP3 inflammasome, thereby reducing platelet survival (86). Tyrosyl-tRNA synthetase (YRSACT) induces the generation of “inflammatory” megakaryocytes and platelets by activating TLR and type I interferon signaling pathways, and these platelets exhibit enhanced agonist-induced activation and pro-coagulant activity (87). Low-grade endotoxemia (LGE) enhances platelet activation and thrombosis formation through the TLR4 signaling pathway, a mechanism of significant importance in cardiovascular diseases (88). Additionally, platelets are involved in the pathological mechanisms of hemolytic disorders by expressing TLR4 and the NLRP3 inflammasome. For instance, free hemin can stimulate the release of α-granules from platelets through the activation of the TLR4 signaling pathway, which in turn activates the production of mitochondrial reactive oxygen species (mtROS) (89). These results offer novel insights for advancing therapies aimed at anti-thrombosis and anti-inflammation that focus on the signaling pathways of platelet TLR.

#### 4.2.3 Monocytes

LPS activates monocytes through TLR4, inducing the transient expression of TF, which triggers the coagulation cascade (90). Anti-phospholipid antibodies (aPL) stimulate monocytes and endothelial cells via TLR2, which activates the NF-κB signaling pathway and enhances the production of pro-inflammatory cytokines (such as TNF-α and IL-6). This process highlights the role of TLR2 in mediating inflammatory responses associated with antiphospholipid syndrome (APS) (91). Similarly, autoantibodies against β2-glycoprotein I (β2GPI) induce a pro-inflammatory phenotype in endothelial cells and monocytes by cross-reacting with TLR4, further elucidating the role of TLR4 in APS pathology (92). Furthermore, the exosomes found in body fluids activate monocytes through the TLR2 and TLR4 signaling pathways, which in turn promotes the secretion of inflammatory cytokines like IL-6 and TNF-α, underscoring the potential role of exosomes in inflammatory diseases (93). In addition to the TLR signaling pathway, the Notch signaling pathway significantly influences monocyte differentiation and inflammatory responses. The TLR7-Myd88 pathway and Notch2 together determine the fate of monocytes. When Notch2 signaling is absent, monocytes may aberrantly differentiate into dendritic cells and macrophages, resulting in an excessive elevation of systemic inflammation. This highlights the crucial role of Notch2 in the differentiation of monocytes mediated by TLR (94). Functional abnormalities of monocytes in pathological conditions can lead to severe clinical consequences.

#### 4.2.4 Macrophages

The activation of TLR9 and TLR7/8 has been shown to induce the formation of multinucleated macrophages, indicating chronic inflammation and granuloma formation. This suggests that TLR signaling is central to macrophage activation and the progression of inflammatory responses (95). Additionally, TLR activation coordinates the mobilization of arachidonic acid in macrophages, a process regulated by group IVA and V phospholipases (96). This mechanism is important for initiating and amplifying inflammatory responses. The TRIM protein family also plays an important role in macrophage activation after TLR stimulation, especially after TLR3 and TLR4 activation, contributing to the regulation of immune responses (97). Moreover, interferon (IFN) has been shown to enhance the expression of TLR genes in macrophages during viral infections, thereby amplifying the innate immune response (98). In addition to pro-inflammatory responses, TLR7 and TLR9 ligands also shift macrophage function to a long-lived phagocytic state, characterized by M2-like polarization and reduced antigen-presenting capacity (99). Chronic signaling through TLR7 and TLR9 is also associated with driving the differentiation of inflammatory hemophagocytic cells, leading to anemia and thrombocytopenia observed in conditions such as macrophage activation syndrome (MAS) and malaria (100). These findings collectively emphasize the key role of TLR signaling in macrophage function, inflammation, and tissue-specific responses in various diseases.

#### 4.2.5 Neutrophils

Neutrophils are the first responders in inflammatory reactions, promoting thrombus formation by releasing reactive oxygen species (ROS) and neutrophil extracellular traps (NETs), which disrupt the endothelial cell barrier. NETs are mesh-like structures composed of chromatin and antimicrobial proteins that can capture platelets and red blood cells, forming the core structure of thrombi (32, 101). TLR signaling pathways influence thrombus formation mechanisms by regulating the formation and stability of NETs. Activation of TLR4 prompts neutrophils to produce NETs, which contain histones and DNA that subsequently stimulate the TLR4 and TLR9 signaling pathways. This generates a positive feedback loop that amplifies inflammatory and coagulation responses, thereby facilitating thrombus formation (102, 103). Additionally, TLR2 promotes NET formation by recognizing bacterial lipopeptides, further increasing thrombus stability (104). Brill et al. (105) demonstrated that NETs play a key role in DVT in mice. They found that extracellular chromatin from neutrophils contributes to thrombus formation. The study further indicated that DNA and histones in NETs are involved in promoting DVT, providing new insights for DVT treatment strategies.

### 4.3 The role of TLR-mediated crosstalk between platelets and leukocytes in DVT

The pathological mechanisms of DVT involve complex biological processes including vascular endothelial injury, platelet activation, and inflammatory cell recruitment, where the cross-talk between platelets and leukocytes plays a pivotal role in disease development. The TLR-mediated interactions between platelets and leukocytes exert critical regulatory effects on DVT pathogenesis. Activated platelets express surface markers such as P-selectin and CD40L, enabling them to bind leukocytes via PSGL-1 and integrins, respectively (106–108). These interactions facilitate the formation of platelet-leukocyte aggregates, thereby enhancing leukocyte recruitment, tissue factor expression, and NET release—key drivers of venous thrombosis (7, 109, 110). In inflammatory microenvironments triggered by infection or tissue injury, DAMPs and PAMPs released from necrotic/apoptotic cells activate TLRs on leukocytes and platelets, establishing a vicious cycle of prothrombotic and proinflammatory responses (111, 112), particularly prominent in severe COVID-19, dengue virus infection, and other inflammatory crises (113, 114). Targeting the TLRs activation pathways may alleviate thrombotic events and hyperinflammatory states, thereby breaking the thrombo-inflammatory vicious cycle.

In sepsis and cardiovascular diseases, platelet TLRs promote leukocyte recruitment and create prothrombotic microenvironments through MyD88 signaling by inducing proinflammatory factors like IL-1β and CD40L (115). Human platelets recognize bacterial components (e.g., Pam3CSK4) via TLR2, activating the PI3-K/Akt pathway to induce platelet-neutrophil aggregation and oxidative stress, potentially contributing to thrombosis risk post-bacterial infection (116). TLR7 activation in platelets triggers p38 MAPK and Akt phosphorylation, leading to α-granule release (e.g., surface expression of P-selectin and CD40L) that promotes platelet-leukocyte aggregation (117). During viral infections, platelets activate TLR-MyD88-IRAK4 cascades through viral particle endocytosis, triggering complement C3 release and platelet-leukocyte aggregation (118). Influenza virus specifically induces C3 secretion and NET formation via platelet TLR7 (119), revealing molecular mechanisms of viral-associated thrombosis. Conversely, histones in NETs activate platelets through TLR2, TLR4 and NLRP3 inflammasomes, promoting P-selectin expression and phosphatidylserine (PtdSer) exposure to enhance coagulation factor binding (120–122). Additionally, extracellular vesicles (EVs) generated by platelet CLEC2 activation drive neutrophil NETosis via CLEC5A/TLR2 dual receptors and stimulate macrophages to secrete IL-6 and TNF-α (123). Platelet-derived HMGB1 facilitates monocyte migration and inhibits apoptosis through the RAGE/TLR4 axis (124), while disulfide HMGB1 from platelets mediates neutrophil recruitment and NET release via RAGE/TLR2 in experimental DVT, establishing a self-amplifying “thrombo-inflammatory loop” (125).

Notably, platelets exert homeostatic regulation on leukocyte functions. TLR2 agonists (e.g., Pam3CSK4 and FSL-1) suppress neutrophil CD66b expression and elastase release while enhancing phagocytosis (126). Platelets modulate cytokine secretion in peripheral blood mononuclear cells (PBMCs) via TLR4 and TLR2 signaling (e.g., reducing IL-6 but increasing IL-10) (127), suggesting TLRs balance pro- and anti-inflammatory responses through spatiotemporal signaling. This regulatory pattern may influence thrombus stability by dynamically modulating local inflammatory milieus in DVT. Furthermore, aberrant platelet-leukocyte interactions can induce sterile inflammation and tissue damage, particularly evident in immune-mediated vascular disorders (128). Therefore, elucidating TLR-mediated platelet-leukocyte cross-talk not only advances understanding of DVT pathogenesis but also provides insights for developing novel therapeutic strategies.

### 4.4 Interactions between TLRs and the coagulation system

The interaction between TLR signaling pathways and the coagulation system is particularly important in the pathophysiology of DVT. TLR activation promotes coagulation responses through various mechanisms, enhancing thrombus formation and stability.

The regulation of TF expression is one of the main mechanisms by which TLR signaling pathways promote coagulation responses. The activation of TLR4 and TLR2 directly induces TF expression, with TF being a key molecule initiating the coagulation cascade. By binding to coagulation factor VIIa, TF activates thrombin, promoting fibrin formation and thrombus development (120, 129). Xia et al. (130) found that TLR4 activation significantly increases TF expression through the NF-κB and AP-1 signaling pathways, further promoting coagulation responses.

The inhibition of anticoagulant factors is also an important pathway by which TLR signaling pathways promote a hypercoagulable state. Activation of TLR4 not only enhances the production of thrombin but also reduces the expression of anticoagulant proteins (such as protein C, protein S, and antithrombin III), resulting in a hypercoagulable environment that facilitates the formation and stabilization of thrombi (131). Biswas et al. (132)found that TLR2 senses oxidized phospholipids binding to CD36 on platelets, activating the MyD88 signaling pathway, thereby leading to a hypercoagulable state and accelerating thrombosis formation associated with hyperlipidemia. Haseeb et al. (133) discovered that TLR9 markedly boosts the generation of pro-inflammatory and coagulation factors through the activation of the MAPK and NF-κB signaling pathways, which in turn facilitates a hypercoagulable condition.

Additionally, TLR9 activation enhances thrombus stability by inducing the production of type I interferons and regulating thrombin generation and fibrin stability (134). These multiple mechanisms collectively indicate that TLR signaling pathways play an important role in the regulation of the coagulation system in DVT.

### 4.5 Downstream effects of TLR signaling pathways and their specific roles in DVT

The activation of TLR signaling pathways regulates the occurrence and development of DVT through multiple downstream effector pathways. The main downstream effector pathways include the NF-κB, MAPK, and IRF signaling pathways, which jointly regulate the expression of inflammatory factors and the enhancement of coagulation responses.

#### 4.5.1 NF-κB signaling pathway

As an essential transcription factor that plays a significant role in TLR signaling pathways, NF-κB is vital for the development of DVT. In a rat model featuring femoral fractures, the activation of TLR2 significantly increased the concentrations of NF-κB, COX-2, along with inflammatory factors like IL-6 and P-selectin. Conversely, the inhibition of TLR2 diminished these effects, underscoring the critical role of the TLR2/NF-κB signaling pathway in the development of thrombotic disease following deep vein thrombosis (135). The multiple roles of the NF-κB signaling pathway in inflammation, immune regulation, and disease have been widely studied, with its interaction with TLR signaling being particularly prominent in thrombosis formation (136). In contrast, cotinine, a substance produced from nicotine metabolism, enhances the development of DVT and inflammatory responses through the TLR4/NF-κB signaling pathway, highlighting the essential role of NF-κB in thrombosis progression (137). At the molecular level, TLR signaling activates NF-κB through the myddosome complex, thereby regulating the expression of inflammatory genes, a mechanism of significant importance in thrombosis formation (138). To delve deeper into the function of NF-κB within TLR-related inflammatory responses, scientists have created a range of approaches, including the measurement of NF-κB’s movement into the nucleus using imaging techniques, which offer valuable resources for investigating its roles in conditions such as DVT (139).

#### 4.5.2 MAPK signaling pathway

The MAPK signaling pathway serves as a crucial downstream effector of TLR signaling and is significant in the development of DVT. Studies have shown that the MAPK signaling pathway contributes to the development and advancement of DVT by affecting the expression of inflammatory mediators and various biological processes, such as cell growth and differentiation. For instance, the p38 MAPK and JNK components of the MAPK signaling pathway enhance the production of pro-inflammatory cytokines such as TNF-α and IL-6 by activating TLR signaling, which are crucial in the inflammatory responses associated with DVT (140). Moreover, the MAPK signaling pathway significantly contributes to inflammation triggered by endoplasmic reticulum stress and NOD2 activation, as its activation intensifies the secretion of inflammatory factors in macrophages (141). Within the MAPK signaling pathway, MAPK-activated protein kinases (MAPKAPKs) serve as downstream effectors and contribute to the progression of chronic inflammatory diseases, such as DVT, by influencing the expression of genes related to inflammation (142). In a mouse model of DVT, Fisetin markedly decreased the levels of pro-inflammatory cytokines and oxidative stress through the inhibition of the MAPK signaling pathway (such as p38 and JNK), thereby reinforcing the crucial function of the MAPK pathway in DVT (143). Additionally, UCHL1 plays a role in the inflammatory response of macrophages stimulated by LPS by modulating the MAPK and NF-κB pathways, highlighting the importance of MAPK signaling in inflammation linked to DVT (144). Finally, the MAPK signaling pathway’s independent regulation of TLR expression and cytokine production from NF-κB further emphasizes its central position in DVT inflammatory responses (145).

#### 4.5.3 IRF signaling pathway

TLR signaling pathways play a key role in immune regulation and inflammatory responses by activating IRF transcription factors (e.g., IRF3 and IRF7). The triggering of type I interferon production (like IFN-β) occurs through TLR signaling, which activates IRF3 and IRF7, thereby regulating both antiviral and inflammatory responses (146). In particular, in sterile inflammatory diseases such as DVT, the TLR9-IRF1 signaling pathway affects thrombus formation and resolution by regulating inflammatory and coagulation responses (147). Additionally, the TLR3 signaling pathway induces the expression of type I interferons by activating IRF3, while certain antipsychotic drugs can inhibit TLR3-mediated inflammatory responses by suppressing the PI3K signaling pathway and inhibiting IRF3 activation (148). The irregular activation of the IRF signaling pathway is closely linked to inflammatory conditions, such as DVT. In this context, IRF3 and IRF7 play a crucial role in the regulation of thrombus formation and its resolution by modulating inflammatory responses and coagulation processes (149, 150).

### 4.6 Cross-regulation and feedback mechanisms of TLR signaling pathways

The involvement of TLR signaling pathways in DVT is evident not only through the activation of specific pathways but also through the interplay and feedback interactions among various signaling routes. Following the activation of TLR4, it triggers not only NF-κB and IRF3 via the MyD88 and TRIF pathways but also plays a role in the immunity and inflammation regulation by influencing additional signaling pathways, such as PI3K/Akt and JAK/STAT (151). These cross-regulation mechanisms enhance the regulatory effects of TLR signaling pathways in DVT and also provide multiple targets for DVT treatment.

#### 4.6.1 PI3K/Akt signaling pathway

The role of the PI3K/Akt signaling pathway is crucial in the regulation of inflammation, cell survival, and metabolic activities, particularly within the framework of TLR signaling. TLR activation (including TLR2 and TLR4) of PI3K/Akt signaling contributes to the regulation of pro-inflammatory cytokines. The expression of TLR4 in macrophages is elevated by hypoxic stress via the PI3K/Akt signaling pathway, which intensifies inflammation associated with ischemic conditions (152). The interplay between the PI3K/Akt/mTOR and TLR/NF-κB signaling pathways is crucial for the response to streptococcal infections. This suggests that TLR2 and TLR4 initiate the activation of the PI3K/Akt/mTOR pathway to control inflammation within mammary epithelial cells (153). Furthermore, the TLR4/PI3K/Akt pathway regulates LPS-induced proliferation of vascular smooth muscle cells (VSMCs), linking this signaling pathway to diseases such as atherosclerosis (154). Collectively, these investigations emphasize the crucial involvement of PI3K/Akt in the modulation of inflammation and immune responses via TLR signaling, indicating possible therapeutic approaches for addressing inflammation across different diseases.

#### 4.6.2 JAK/STAT signaling pathway

The JAK/STAT signaling pathway plays a key role in immune regulation, especially exhibiting important regulatory functions in its interaction with TLR signaling pathways. STAT1 plays a significant role in TLR signaling, particularly after TLR4 activation, where STAT1 enhances TLR-mediated inflammatory responses through interactions with TRAF6 (155). The study showed that TLR3 stimulation could “pre-activate” macrophages, thereby enhancing the immune response to subsequent TLR7 stimulation and promoting the synergistic production of cytokines through the JAK-STAT pathway (156). The crosstalk between JAK/STAT, TLR, and ITAM-dependent pathways in macrophages was also further explored, with the study showing that IFN-γ promotes TLR-mediated immune responses by modulating the interactions among these signaling pathways (157). At the same time, the signaling pathways of TLR work in conjunction with JAK/STAT signaling via cytokines like IFN-γ and IL-10, offering fresh perspectives on the intricate regulatory network of immune system signaling (158).

### 4.7 Negative regulatory mechanisms of TLR signaling pathways

To avoid excessive stimulation of TLR signaling pathways and mitigate uncontrolled inflammatory responses, the body employs various negative regulatory mechanisms. These mechanisms inhibit key nodes of signal transduction, limiting the intensity and duration of inflammatory responses to maintain immune balance.

#### 4.7.1 A20 protein

A20 (TNFAIP3) functions as an important negative regulator, playing a significant role in modulating the NF-κB signaling pathway during immune responses. It regulates the intensity and duration of inflammatory responses by inhibiting ubiquitination modifications in TLR signaling pathways through its ubiquitin-editing functions. A20 directly affects the activation process of NF-κB through deubiquitination, ensuring the timely termination of immune responses (159). A20 interacts with E3 ligases such as TRAF6 to prevent the activation of these signaling molecules in TLR signaling. For example, Düwel et al. (160) discovered that A20 prevents the prolonged activation of NF-κB through the removal of K63-linked ubiquitin chains from Malt1, an action essential for the correct functioning of immune cells. Shembade et al. (161) further demonstrated that A20 inhibits the excessive activation of TLR signaling pathways by disrupting the E3 ligase activities of TRAF6, TRAF2, and cIAP1, thereby avoiding sustained inflammatory responses.

#### 4.7.2 SOCS family proteins

Emerging evidence has identified Suppressors of Cytokine Signaling (SOCS) proteins as key regulators of TLR signaling networks. These cytokine-induced proteins act as molecular rheostats, maintaining immune homeostasis through negative feedback mechanisms activated by cytokine receptors and pattern recognition receptors, including TLRs themselves. Specific SOCS family members (SOCS1–3 and CIS) differentially regulate TLR signaling by selectively targeting interferon pathways while preserving NF-κB activation, enabling precise control of antiviral responses without compromising inflammatory signaling (162). SOCS proteins show functional specialization in different immune cells, with SOCS1 in myeloid cells like monocytes and macrophages specifically limiting IFN - dependent signaling (163). Recent advances highlight SOCS1 and SOCS3’s dual-layer regulation of the TLR7 pathway in human plasmacytoid dendritic cells, balancing antiviral responses and immune tolerance through IRF7 degradation (164). This complex regulatory paradigm highlights the context-dependent nature of SOCS protein functions, where the cellular microenvironment, receptor crosstalk, and temporal expression patterns collectively determine their immunomodulatory outcomes.

### 4.8 The role of TLRs in DVT resolution

Early murine model studies demonstrated that the TLR9 signaling pathway facilitates early thrombus resolution by regulating sterile inflammatory responses (including inflammatory cell infiltration, thrombus collagenization, and necrotic pathways), with TLR9 knockout mice exhibiting significantly increased thrombus volumes (134). Further investigations revealed that TLR9 deficiency impairs macrophage function, manifesting as reduced chemotactic capacity and downregulated fibrinolytic gene expression, while exogenous transfusion of wild-type macrophages effectively restored thrombus resolution capability (165). In neutrophils, TLR9 influences early venous thrombus formation through modulation of necrotic markers such as uric acid and NETs (166). Clinical studies showed that although TLR9 mRNA expression in DVT patients showed no significant correlation with thrombus remnants, it was markedly elevated in recurrent DVT cases, suggesting TLR9 may possess a dual regulatory role while also indicating potential mechanistic differences in TLR9 regulation between animal models and human clinical samples (167). Similar to TLR9’s mechanism, TLR4 deficiency was found to inhibit thrombus dissolution by reducing neutrophil/macrophage infiltration and downregulating MMP-9/MCP-1 expression, a process dependent on the NF-κB signaling pathway, thereby expanding understanding of TLR family roles in thrombus clearance (168). Current studies propose a two-phase immune model of thrombus resolution: an early phase where neutrophils stabilize thrombus structure through NETs, followed by a macrophage-dominated fibrotic and endothelialization phase (169). In summary, TLRs play multifaceted roles in resolving deep vein thrombosis by modulating inflammation, interacting with multiple signaling pathways, and influencing immune cell activities including macrophages. Understanding the mechanisms by which TLRs promote thrombus dissolution may facilitate development of novel therapeutic strategies aimed at enhancing DVT resolution while reducing thromboembolic complication risks (170).

## 5 The potential of TLR signaling pathways as therapeutic targets for DVT

Therapeutic strategies targeting TLR signaling pathways primarily focus on suppressing excessive TLR activation or blocking downstream signaling to mitigate acute-phase inflammatory and coagulation responses, thereby preventing early-stage inflammatory thrombus formation (**Table 2**).

**Table 2:** Drugs associated with TLR signaling pathways.

Drugs	Target	Mechanism of Action	References
C29	Inhibits TLR2	Interfere with the recruitment of adaptor proteins such as MyD88 and Mal	(135, 171, 172)
SMU-CX1	Inhibits TLR3	Specifically inhibit the binding of TLR3 with dsRNA	(212)
Eritoran	Inhibits TLR4	Competitively bind to the TLR4-MD-2 complex, blocking the binding of LPS and the activation of TLR4	(173–175)
Enpatoran	Inhibits TLR7 and TLR8	Block the binding of synthetic ligands and natural endogenous RNA ligands to TLR7 and TLR8, thereby inhibiting the activation of the receptors	(213)
E6446	Inhibits TLR9	Block the activation of TLR9 induced by its ligands	(214)
TAK-242	Inhibits TLR4	Specifically inhibit the interaction between TLR4 and its adaptor molecules (such as TIRAP and TRAM)	(177)
MiR-149-3p	Inhibits TLR4	Targets the 3′ untranslated region (3′UTR) of TLR-4 mRNA	(215, 216)
MiR-129-5p	Inhibits TLR3	Reduced the expression of IL-1β and TNF-α by inhibiting the HMGB1 and TLR3	(217)
NI-0101	Inhibits TLR4	Inhibiting LPS-induced cytokine release and preventing the increase of C-reactive protein (CRP)	(218, 219)
MTS510	Inhibits TLR4	Binding to the TLR4/MD-2 complex, it blocks the interaction between the complex and its ligands	(220)
Artesunate	Inhibits TLR4	Binds to the TLR4 co-receptor MD2, disrupting its interaction with TLR4	(221)
Flavonoids	Inhibits TLR4	Suppress TLR4/MyD88 signaling, reducing IL-6 and VCAM-1 release in endothelial cells exposed to hypoxia.	(222)
Phillygenin	Inhibits TLR4	Inhibiting the TLR4/MyD88/NF-κB signaling pathway	(187)
Sinomenine	Inhibits TLR4	Inhibiting the TLR4/MyD88/NF-κB signaling pathway	(189)
Baicalin	Inhibits TLR4	Block the activation of the LPS-induced TLR4/NF-κB p65 signaling pathway and inflammatory response by inhibiting the expression of CD14.	(210)

### 5.1 TLR inhibitors/antagonists

TLR inhibitors/antagonists, by inhibiting TLR activation, may effectively alleviate the inflammatory response during thrombosis and reduce the incidence of DVT.

C29, which selectively inhibits TLR2, is able to obstruct the activation of downstream signaling pathways by preventing TLR2 from binding with its ligands. Research indicates that C29 interferes with the interaction between TLR2 and its adaptor protein MyD88 by binding to the BB loop pocket located in the TIR domain of TLR2, which prevents the activation of downstream signaling pathways involving MAPK and NF-κB (171). Subsequent investigations revealed that C29 effectively diminishes the generation of inflammatory substances by blocking NF-κB activation facilitated by TLR2/1. When combined with the analog MMG-11, it exhibits a synergistic effect that further amplifies the suppression of TLR2 signaling (172). Recent research has shown that C29 markedly decreases the expression of inflammatory markers, including IL-6 and TNF-α, through the inhibition of the TLR2/NF-κB/COX-2 signaling pathway, which helps relieve traumatic deep vein thrombosis (135). These studies collectively suggest that C29, as a TLR2 inhibitor, has broad application potential in various diseases involving inflammation and thrombosis.

Eritoran, which is an artificial antagonist of TLR4, has the ability to competitively attach to the TLR4-MD-2 complex, preventing LPS from binding and inhibiting TLR4 activation (173). However, despite showing significant TLR4 inhibitory effects *in vitro*, its clinical application has been limited. A multicenter randomized controlled trial showed that Eritoran did not significantly reduce the mortality rate of patients with severe sepsis, but it had good safety (174). On the other hand, in a mouse model of influenza virus infection, Eritoran significantly reduced pulmonary inflammatory responses and viral loads by inhibiting the TLR4 signaling pathway, indicating its potential application value in treating influenza-related acute lung injury (175). Recently, there has been considerable discourse surrounding Eritoran’s potential in addressing inflammation, infections, and cancer. Additionally, innovative approaches for regulating TLR4, utilizing disaccharide lipid A analogs, have been suggested, offering fresh avenues for enhancing and applying TLR4 antagonists in clinical settings (176).

TAK-242 (Resatorvid), a small molecule TLR4 inhibitor, selectively binds to the intracellular TIR domain of TLR4, blocking downstream signal transduction and thereby reducing the production of inflammatory factors. Initial research indicated that TAK-242 had the capability to selectively block the interaction of TLR4 with its adaptor proteins (including TIRAP and TRAM), leading to a marked decrease in the synthesis of inflammatory mediators triggered by LPS (like TNF-α and IL-6) and hindering the activation of NF-κB (177). Further research found that the specific inhibitory effect of TAK-242 on TLR4 was independent of other TLRs (such as TLR1/2, TLR3, etc.), indicating its high selectivity (178). Recent studies have shown that TAK-242 significantly reduces the production of inflammatory mediators, such as IL-1β, IL-6, and TNF-α, in synovial fibroblasts derived from patients with osteoarthritis. This effect is achieved by inhibiting the TLR4-NF-κB signaling pathway (179). These studies collectively suggest that TAK-242, as a TLR4 inhibitor, has broad application potential.

### 5.2 MicroRNAs

MicroRNAs (miRNAs) are small non-coding RNAs that serve important regulatory functions in a range of biological processes. In recent years, the role of microRNAs in TLR signaling pathways and DVT has gradually become a research hotspot. MiRNAs influence TLR signaling by targeting key components of the pathway. For instance, miR-146a and miR-155 are known to regulate the expression of proteins involved in TLR signaling, thereby modulating the immune response (180). A rat model demonstrated the expression patterns of miRNAs and mRNAs in DVT, identifying 22 miRNAs and 487 mRNAs that were significantly different between the DVT group and controls, primarily associated with endothelial cell activity. This suggests that miRNAs are crucial in the development of thrombosis through their regulatory influence on endothelial cells (181). Studies concerning human hemophilia and thrombosis have demonstrated that miRNAs significantly influence thrombosis development by regulating the expression of coagulation and inflammatory mediators, thereby providing a new perspective for the diagnosis and treatment of thrombotic conditions (182). Recent studies have discovered that miRNAs significantly influence the development of DVT by modulating processes like cell growth, differentiation, the release of inflammatory factors, and vascular remodeling. This offers a foundational understanding that supports the use of miRNAs in diagnosing and treating DVT (183). Understanding the roles of miRNAs in TLR signaling and DVT not only provides insights into the molecular mechanisms underlying these conditions but also offers potential targets for therapeutic interventions.

### 5.3 Monoclonal antibodies

Monoclonal antibodies targeting TLRs are also an effective inhibitory approach. Anti-TLR4 monoclonal antibodies, by specifically blocking the TLR4 signaling pathway, have shown significant therapeutic potential in various inflammatory and injury-related diseases. The Fab fragment derived from a humanized anti-TLR4 monoclonal antibody was discovered to effectively suppress inflammatory responses induced by LPS (184). This effect is achieved by preventing the interaction of TLR4 with its ligand, which leads to a marked decrease in the activation of both NF-κB and MAPK signaling pathways. Consequently, this action inhibits the release of cytokines, thereby offering a theoretical foundation for therapeutic strategies targeting TLR4 that utilize the Fab fragment. Additionally, anti-TLR4 monoclonal antibodies were found to significantly alleviate ventilator-induced lung injury (VILI) by inhibiting MyD88 and NF-κB-dependent signaling pathways, reducing pulmonary inflammatory cell infiltration and cytokine release, and improving pathological damage to lung tissue (185). In recent years, the development of a fully human anti-TLR4 IgG2 antibody has further expanded research in this area. This antibody demonstrated a high affinity for TLR4 *in vitro*, successfully reducing the levels of TNF-α, IFN-β, and IL-6 that were triggered by LPS, while simultaneously diminishing the phosphorylation of the NF-κB, MAPK, and IRF3 signaling cascades. Notably, in animal studies, the antibody greatly enhanced the survival rate of mice subjected to an LPS challenge (186). Collectively, these studies suggest that monoclonal antibodies targeting TLR4 hold significant potential for application across different disease models, offering novel insights for the clinical management of associated ailments.

### 5.4 Natural products and herbal

Natural products regulate TLR signaling pathways through various mechanisms and have good anti-inflammatory and anticoagulant effects. Natural products serve as significant potential drugs and offer a theoretical foundation for developing novel strategies to treat DVT.

Phillygenin, a lignan compound derived from the Forsythia suspensa fruit, exhibits notable anti-inflammatory, antioxidant, and neuroprotective properties. In recent years, its pharmacological activities in various disease models have attracted widespread attention, especially its potential therapeutic value in inflammation-related diseases and central nervous system diseases. Phillygenin can effectively inhibit LPS-induced activation and inflammatory response of LX2 cells by inhibiting the TLR4/MyD88/NF-κB signaling pathway, indicating its potential therapeutic value in hepatic fibrosis and related inflammatory diseases (187). In the field of neuroprotection, Phillygenin can reduce neuroinflammatory responses after spinal cord injury and promote functional recovery by inhibiting the TLR4/NF-κB signaling pathway, providing a scientific basis for its application in central nervous system diseases (188). These studies collectively reveal the multiple mechanisms of action of Phillygenin and Forsythia-related components in anti-inflammatory and neuroprotective effects, laying a theoretical foundation for their further development into clinical drugs.

Sinomenine (SIN), a alkaloid extracted from the Chinese medicine Sinomenium acutum, has significant anti-inflammatory, immunomodulatory, and neuroprotective effects. Recently, there has been significant interest in its potential use for treating inflammatory and autoimmune diseases. Studies show that Sinomenine exhibits significant anti-inflammatory and immunomodulatory effects across various disease models by influencing the TLR4/NF-κB signaling pathway (189). The investigation of rheumatoid arthritis (RA) revealed that Sinomenine markedly diminished the inflammatory reaction of fibroblast-like synoviocytes (FLS) while also decreasing the levels of MyD88 and TRAF6 by blocking the TLR4/MyD88/NF-κB signaling pathway, thereby offering molecular support for the treatment of RA (190). Recent research indicates that Sinomenine contributes to the equilibrium between the brain and gut through the modulation of the cholinergic anti-inflammatory pathway (CAP) and the inhibition of the TLR4/NF-κB signaling pathway in a mouse model of Alzheimer’s disease (AD) (191). This results in significant improvements in cognitive abilities and a decrease in inflammatory responses.

Baicalin, a flavonoid derived from the roots of Scutellaria baicalensis, exhibits notable anti-inflammatory, antioxidant, and immunomodulatory properties. In recent years, its role in regulating TLR signaling pathways and related inflammatory diseases has attracted much attention. Research indicates that baicalin may inhibit the activation of the TLR4/NF-κB p65 signaling pathway triggered by LPS, as well as the related inflammatory response, through the suppression of CD14 expression (192). Moreover, the findings indicated that baicalin has the potential to reduce joint inflammation and tissue damage in a rat model of collagen-induced arthritis (CIA) by modulating the TLR2/MYD88/NF-κB p65 signaling pathway, thus offering a novel therapeutic approach for rheumatoid arthritis management (193). Recent research has expanded the range of applications for baicalin, showcasing its significant anti-inflammatory and antioxidant effects in various inflammatory ailments such as hepatitis, rheumatoid arthritis, obesity, and type 2 diabetes by intervening in the TLR/NF-κB signaling pathway (194).

### 5.5 Combination therapy

In recent years, combination therapies have demonstrated remarkable potential in inflammatory regulation and organ protection, particularly showing significant advancements in synergistic suppression of immune signaling pathways (e.g., TLR) and multi-mechanism interventions. Multiple clinical studies have confirmed that the combination of antiplatelet therapy (Triflusal) with moderate-intensity anticoagulation (Acenocoumarol) reduces vascular event risk by 67% in moderate-to-high risk atrial fibrillation patients without significantly increasing major bleeding rates (195). This combined strategy shows greater efficacy in mechanical heart valve patients, achieving a 35% relative risk reduction in thrombotic events, though requires cautious selection in atrial fibrillation populations based on individual bleeding risks (196). Basic research reveals that the protease inhibitor Bortezomib (VELCADE) combined with tPA suppresses the TLR4/IRAK1 pathway in endothelial cells via miR-146a upregulation, reducing cerebral infarct volume by 46% in rat stroke models while reversing tPA-induced vascular inflammation (197). In oncology and surgical fields, the TLR4 inhibitor TAK-242 combined with estrogen antagonist Fulvestrant inhibits non-small cell lung cancer metastasis (198), while a compound formulation of TAK-242 with hyaluronic acid reduces postoperative abdominal adhesion incidence by 64% in mice through TLR4/ROS signaling inhibition (199). Notably, the combination of Pentoxifylline (PTX) and Dexamethasone demonstrates synergistic suppression of TLR4/7/8 pathways, showing 3.2-fold greater efficacy in neonates than adults through bidirectional regulation of TNF/IL-10 mRNA balance (200). Novel drug delivery systems like omeprazole nanoliposomes achieve targeted inhibition of endosomal TLR3/4/7/8 signaling in macrophages, validating the translational value of “nanocarrier + drug repurposing” strategies (201). These findings align with the critical role of dynamic TLR pathway modulation, exemplified by TAK-242 combined with G-CSF reducing mortality from 66% to 0% in ACLF mouse models (202), further highlighting the broad prospects of combination therapies.

## 6 Discussion

Although TLR signaling pathways have significant potential as therapeutic targets for DVT, their clinical application still faces many challenges. First, the key role of TLR signaling pathways in immune responses means that their inhibitors may suppress immune function and increase the risk of infection (9). Therefore, the development of TLR inhibitors with high selectivity and low toxicity is one of the current research priorities. Second, the complexity and diversity of TLR signaling pathways mean that the inhibition of a single target may not comprehensively suppress the enhanced inflammatory and coagulation responses, affecting therapeutic effects (203). Moreover, the polymorphism of TLR genes and differences in epigenetic regulation among individuals may also affect the therapeutic effects and safety of TLR inhibitors, requiring the support of personalized treatment strategies (204). Finally, regarding the dual role of TLR signaling: TLR activation can both exacerbate thrombosis (e.g., through pro-inflammatory factor release) (205, 206) and promote DVT resolution (e.g., by regulating macrophage polarization) (165, 207), with precise regulatory strategies remaining to be explored.

TLR signaling pathways play a key role in the inflammatory response of DVT. In-depth studies of their regulatory mechanisms provide important directions for the development of new therapeutic strategies. The development of more specific TLR inhibitors through high-throughput screening and structural optimization to precisely regulate inflammatory responses. For example, studies based on molecular dynamics simulations and molecular docking have found that the Arg241 residue of TLR4 and the Tyr102, Ser120, and Lys122 residues of MD2 are key sites for antagonist ligand binding, providing important information for structure-based TLR4 inhibitor design (208, 209). TLR signaling pathways interact with other signaling pathways such as PI3K/Akt and JAK/STAT, which are involved in the inflammatory response of DVT (210, 211). However, a thorough assessment of potential adverse effects (particularly hemorrhagic complications) is warranted to ensure clinical safety. Therefore, the development of multi-target drugs may more effectively treat DVT and reduce the occurrence of drug resistance. Finally, in-depth studies of the molecular mechanisms of TLR signaling pathways, especially the formation and dynamic changes of the myddosome, will help reveal their role in DVT. For example, recent studies have shown that the myddosome, as the core platform of TLR signal transduction, is crucial for the initiation and maintenance of inflammatory responses through its assembly and functional regulation (138).

## 7 Conclusion and outlook

In conclusion, the signaling pathways associated with TLR are crucial in the development of DVT. They facilitate the amplification of inflammatory and coagulation reactions via multiple mechanisms, thereby promoting thrombus formation and stabilization. Members of the TLR family, such as TLR4, TLR2, and TLR9, induce the expression of inflammatory and coagulation factors by activating downstream MyD88-dependent and independent signaling pathways. This process promotes the engagement and activation of immune cells, consequently enhancing the development and stability of blood clots. Moreover, TLR signaling pathways further promote thrombus formation and expansion through their interaction with vascular endothelial cells and the coagulation system. The sources of endogenous and exogenous TLR ligands in DVT provide an important driving force for the activation of TLR signaling pathways. Additionally, the negative regulatory mechanisms of TLR signaling pathways play a crucial role in maintaining immune balance and limiting inflammatory responses. The improper regulation of TLR signaling pathways may result in heightened activation, facilitating the onset and progression of DVT.

Based on these mechanisms, therapeutic strategies targeting TLR signaling pathways hold important clinical application potential. A variety of drugs, including TLR antagonists, inhibitors, as well as natural products, can effectively inhibit inflammatory and coagulation responses by regulating key molecules and steps in TLR signaling pathways, reducing thrombus formation and stability. Multi-target combined treatment strategies, by simultaneously regulating multiple key molecules and steps, further enhance therapeutic effects and reduce the occurrence of drug resistance, providing new ideas and methods for the treatment of DVT. However, the complexity and diversity of TLR signaling pathways, as well as their key role in immune responses, remain the main challenges for their clinical application. Future research needs to further explore the distinct regulatory mechanisms and interrelationships of TLR signaling pathways, develop highly selective and low-toxicity TLR inhibitors, formulate personalized treatment strategies, and verify their safety and effectiveness through clinical trials, in order to promote the application and translation of TLR signaling pathway-related therapeutic strategies in DVT.
